# Insectivorous birds consume an estimated 400–500 million tons of prey annually

**DOI:** 10.1007/s00114-018-1571-z

**Published:** 2018-07-09

**Authors:** Martin Nyffeler, Çağan H. Şekercioğlu, Christopher J. Whelan

**Affiliations:** 1 Section of Conservation Biology, Department of Environmental Sciences, University of Basel, CH-4056 Basel, Switzerland; 2 Department of Biology, University of Utah, Salt Lake City, UT 84112 USA; 3 College of Sciences, Koç University, Rumelifeneri, Istanbul, Sariyer Turkey; 4 Department of Biological Sciences, University of Illinois at Chicago, Chicago, IL 60607 USA

**Keywords:** Arthropods, Avifauna, Breeding season, Global impact, Insect pests, Predation

## Abstract

In this paper, we present an estimate of the predation impact of the global population of insectivorous birds based on 103 (for the most part) published studies of prey consumption (kg ha^−1^ season^−1^) of insectivorous birds in seven biome types. By extrapolation—taking into account the global land cover of the various biomes—an estimate of the annual prey consumption of the world’s insectivorous birds was obtained. We estimate the prey biomass consumed by the world’s insectivorous birds to be somewhere between 400 and 500 million metric tons year^−1^, but most likely at the lower end of this range (corresponding to an energy consumption of ≈ 2.7 × 10^18^ J year^−1^ or ≈ 0.15% of the global terrestrial net primary production). Birds in forests account for > 70% of the global annual prey consumption of insectivorous birds (≥ 300 million tons year^−1^), whereas birds in other biomes (savannas and grasslands, croplands, deserts, and Arctic tundra) are less significant contributors (≥ 100 million tons year^−1^). Especially during the breeding season, when adult birds feed their nestlings protein-rich prey, large numbers of herbivorous insects (i.e., primarily in the orders Coleoptera, Diptera, Hemiptera, Hymenoptera, Lepidoptera, and Orthoptera) supplemented by spiders are captured. The estimates presented in this paper emphasize the ecological and economic importance of insectivorous birds in suppressing potentially harmful insect pests on a global scale—especially in forested areas.

**Electronic supplementary material:**

The online version of this article (10.1007/s00114-018-1571-z) contains supplementary material, which is available to authorized users.

## Introduction

Birds, represented by nearly 10,700 species, are found across the world in all major terrestrial biomes. Accordingly, they exhibit a large variety of life styles and foraging behaviors (see Wiens 1989). While some birds depend predominantly on plant diets, such as seeds, fruits, and nectar, others feed as carnivores on animal prey, or as omnivores on a mixed diet of plant/animal matter. Most bird species are insectivores that depend for the most part on insects as prey (Losey and Vaughan 2006; Şekercioğlu 2006a). In this paper, “insectivorous birds” are defined in a wider sense as the total of all bird groups that include, at least temporarily, a considerable percentage of arthropods (in particular insects and spiders) in their diets (Lopes et al. 2016). Included in this definition are also omnivorous birds such as starlings (Sturnidae) and thrushes (Turdidae) that consume large amounts of arthropods in addition to other types of food (Del Hoyo et al. 2016). The predominance of insectivory as a feeding style among birds might be explained by the fact that insects (dominating the land biota in terms of numbers, biomass, and diversity) constitute the largest food base for terrestrial carnivorous animals. So, for instance, social insects alone are assumed to have a standing biomass of > 700 million tons globally (compare Hölldobler and Wilson 1994; Sanderson 1996).

Şekercioğlu (2006b) states that birds are “important but ecologically little known actors in many ecosystems.” Likewise, Wenny et al. (2011) state “Birds provide many ecosystem services, which by and large are invisible and underappreciated.” It has thus been suggested that “quantifying the services provided by birds is crucial to understand their importance for ecosystems and for the people that benefit from them” (Whelan et al. 2015). While several attempts had been undertaken to quantify the food consumption of marine birds and shorebirds on a global scale (e.g., Wiens 1989; Brooke 2004), the combined predation impact of the world’s insectivorous birds is still unknown.

Here, we provide estimates for the annual biomass of prey that is consumed by the global population of insectivorous birds in individual biome types and worldwide based on data from the literature. Furthermore, we present an estimate of the standing biomass of the global population of insectivorous birds. This study is intended as a continuation of the papers by Nyffeler (2000) and Nyffeler and Birkhofer (2017)—who were studying spiders—to get a better understanding of the global extent to which potentially harmful herbivorous insects are suppressed by major natural enemies.

## Methods

### Estimate of the standing biomass of the global population of insectivorous birds

For each of seven terrestrial biome types, the bird biomass across the entire biome was assessed by calculating the product of (*D*) × (*W*) × (*Y*), whereby *D* = mean bird density (birds ha^−1^), *W* = mean bird body mass (kg fresh weight bird^−1^), and *Y* = area size of the entire biome type (ha). The mean breeding bird densities (representing global averages) for the various biome types were extracted from a world literature review by Gaston et al. (2003) and are largely in agreement with North American breeding bird densities compiled by Terborgh (1989, page 71). Area sizes for the various biomes were taken from Saugier et al. (2001); essentially, these values do not differ very much from the more up to date 2010 land cover distribution data provided by FAO (https://ourworldindata.org/land-cover) but are more suitable for our purposes because they are broken down into more detailed cover classes than the latter ones allowing a more rigorous assessment. To obtain a mean body mass for Arctic tundra birds, an overall mean for 18 tundra-inhabiting species (see Sokolov et al. 2012) was calculated based on data from Del Hoyo et al. (2016). An overall mean body mass for desert birds was calculated based on weight data for 26 species occurring in Chihuahuan deserts (Gutzwiller and Barrow 2002). Mean bird body mass values for the remaining six biome types were gathered from the following literature sources: Howell (1971); Karr (1971); Wiens (1973); Holmes and Sturges (1975); Wiens and Nussbaum (1975); Kartanas (1989); and Terborgh et al. (1990).

Summing up the seven subtotals produced an estimate of the standing biomass of the global terrestrial avifauna. From this, an estimate of the standing biomass of the global population of insectivorous birds was deduced, assuming that ≈ 90% of the terrestrial bird individuals in the temperate, boreal, and arctic zones and ≈ 60% in the tropics are arthropod-eaters (see Assumption 1, “Methods” section).

### Estimate of the annual prey consumption of the global population of insectivorous birds

We used a simple model involving few assumptions as is advised in cases where a field of study is still largely undeveloped (Weathers 1983; Nyffeler and Birkhofer 2017). Our estimate is based on mean values of prey consumption ha^−1^ year^−1^ in the various biome types, which subsequently were extrapolated on a global scale. To retrieve comparable data, all values obtained from the literature were converted to kg fresh weight ha^−1^ year^−1^. A total of 103 prey consumption values were gathered from three different information sources:

- Source 1: In 26 cases, published values of prey consumption were used (see Supplementary material).
- Source 2: In 53 cases, energy demand estimates for bird communities extracted from the scientific literature (see Supplementary material) were converted into food consumption measures. The conversions are based on an overall average water content of arthropod prey of ≈ 70% (Zandt 1997; Brodmann and Reyer 1999; Bureš and Weidinger 2000), an energy density of animal matter of 22.5 kJ g^−1^ dry weight (Schaefer 1990), and 75% assimilation efficiency (Wiens 1989). For details see Supplementary material.
- Source 3: There is a lack of data regarding the food consumption rates of bird communities in desert and Arctic tundra biomes. We thus calculated food consumption rates for bird communities in these two biome types based on estimates of daily energy expenditure and breeding bird densities. Energy expended for standard metabolism (*M*, in kcal day^−1^) was calculated with the equation *M* = 129 *W*^0.724^ of Lasiewski and Dawson (1967), whereby *W* equals the weight of an average sized bird in kg. Energy expended under field conditions equals approximately 2.5 times standard metabolism (Holmes and Sturges 1975). For the calculation of the desert biome values, cactus wren (*Campylorhynchus brunneicapillus*, average body mass = 38.9 g; Dunning 2007) was chosen as a standard bird representing this biome type, assuming a breeding season length of 90–180 days (mean = 135 days) for deserts (Wiens 1991). In the case of the Arctic tundra biome, snow bunting (*Plectrophenax nivalis*, average body mass = 42.2 g; Dunning 2007) was used as a standard tundra bird, whereby a breeding season length of ≈ 100 days for the Arctic tundra biome was assumed (Weiner and Głowaciński 1975). By multiplying the resulting energy consumption value for a standard bird with corresponding breeding density values taken from the literature (deserts: Austin 1970; Arctic tundra: Watson 1963; James and Rathbun 1981; Montgomerie et al. 1983; Sokolov et al. 2012), rough estimates of the energy consumption for desert and Arctic tundra bird communities, respectively, during the breeding season were obtained. Subsequently, these energy consumption values were converted into food consumption rates (the same conversion factors being applied as in the previous paragraph), which yielded 18 values for desert and 6 values for Arctic tundra sites. For details see Supplementary material.

The 103 prey consumption values were assigned to the following seven groups of terrestrial biomes: (1) tropical forests, (2) temperate and boreal forests, (3) tropical grasslands and savannas/Mediterranean shrubland, (4) temperate grasslands (incl. meadows, pastures, old fields), (5) cropland, (6) deserts, and (7) Arctic tundra. The data were pooled by computing an average prey consumption value (*x̅* kg ha^−1^ year^−1^) for each biome type. By multiplying the average prey consumption ha^−1^ year^−1^ with the corresponding area size of each biome type (based on Saugier et al. 2001), a prey consumption subtotal for each biome type was derived. Summing up the seven subtotals produced an estimate of the global annual prey consumption by the insectivorous avifauna (Table 2). This figure refers exclusively to arthropod prey, whereas other types of invertebrates, such as earthworms, slugs, and snails, are not included.

Our assessment is based on the following assumptions:

- Assumption 1: Prey consumption measures presented in the literature for land bird communities were downsized to the corresponding values for insectivorous birds, taking into account that an estimated 90% of all land bird individuals (and about two thirds of all species) in the temperate, boreal, and arctic zones are insectivores during the breeding season, whereas ≈ 60% of all individuals (and 62% of all species) in the tropics are insectivores. The figure of 60% has also been chosen for non-tropical desert habitats (see Supplementary material). The figure of 90% for the Palearctic birds has been calculated based on population size/diet composition data for 422 bird species presented in the data base “Birds of Switzerland” of the Swiss Ornithological Institute Sempach; it can be considered to be representative for the European temperate/cold regions (see http://www.vogelwarte.ch/en/birds/birds-of-switzerland/). A similarly high proportion of all breeding land bird individuals in the Nearctic realm are insectivores (calculated based on data presented by Wiens 1973; Wiens and Nussbaum 1975, Holmes et al. 1986; and others). The figure of ≈ 60% for tropical birds is a rough estimate based on various sources (see Karr 1971, 1975; Poulin et al. 1994; Poulin and Lefebvre 1996; Leigh 1999; Sakai 2002; Tscharntke et al. 2008; Maas et al. 2015; Sam et al. 2017).
- Assumption 2: The breeding season diets of the avifauna in temperate forests and in some temperate grasslands are composed of ≥ 75% arthropods (Głowaciński et al. 1984) and those in agricultural areas of ≈ 95% arthropods (Jenny 1990; Jeromin 2002; Gilroy et al. 2009). The diets of desert birds are made up, on average, of ≈ 85% arthropods (e.g., Beal 1907). Accordingly, the food consumption values for insectivorous birds of these biomes were multiplied by a factor of 0.75, 0.95, and 0.85, respectively, to obtain arthropod consumption measures (kg fresh weight ha^−1^ season^−1^). See Supplementary material for exceptions.
- Assumption 3: The arthropod consumption measures for tropical biomes relate to annual totals (breeding season plus non-breeding season; see Karr (1975); Leigh and Smythe (1978); Reagan and Waide (1996); Robinson et al. (2000); Sakai (2002)). By contrast, the arthropod consumption values for temperate biomes available in the literature in most cases constitute exclusively breeding season values. The majority of birds in temperate forests, grasslands, and croplands as well as deserts and Arctic tundra sites are primarily dependent on arthropod prey while feeding their young during the breeding season (see Wiens 1973, 1977; Jenny 1990; Buckingham et al. 1999; Jeromin 2002; Gilroy et al. 2009). Once the breeding season is over, many insectivorous birds leave their temperate/cold zone breeding sites to migrate to warmer areas, resulting in strongly reduced bird densities in the breeding habitats during the non-breeding season (Holmes and Sturges 1975; Karr 1975; Marone 1992; Scebba 2001). At the same time, the vast majority of non-migratory residents, which inhabit temperate/cold zone habitats, switch to a diet made up largely of plant matter during the non-breeding season (Clements and Shelford 1939; Brown et al. 1979; Robinson and Sutherland 1997; Buckingham et al. 1999; Renner et al. 2012). Reduced arthropod consumption by non-migratory birds might be explained by the reduced availability of arthropod prey during the non-breeding season and by the fact that vast regions located in temperate, boreal, and polar climates are covered with a blanket of snow for several months, making foraging for arthropod prey difficult at this time. Notwithstanding that, arthropod consumption in these biomes during the non-breeding season continues to a limited extent (Bruns 1960; Davies 1976; Williams and Batzli 1979; Heinrich and Bell 1995; Kirk et al. 1996; Michalek and Krištín 2009; Vel’ký et al. 2011). We assume that in vast areas of the temperate and cold regions, the arthropod consumption ha^−1^ during the entire non-breeding season is ≈ 5–10% of the breeding season value (see Holmes and Sturges 1973, 1975; Rotenberry 1980a,b; Donald et al. 2001). Therefore, we multiplied the breeding season values for temperate biomes and deserts by 0.075 to obtain the corresponding non-breeding season values.
- Assumption 4: Mediterranean shrublands were classified under “tropical savannas and grasslands” because net primary production and bird densities in these two habitat types are similar (Gaston et al. 2003; Chapin et al. 2011). It must be added that the area size of Mediterranean shrublands is small (280 × 10^6^ ha) relative to the global terrestrial area, and a possible error resulting from insufficient data is likely minor.
- Assumption 5: The estimates presented in this paper are based on studies mostly conducted in the last three decades of the twentieth century. Patterns of bird population decline as discussed more recently (Şekercioğlu et al. 2002, 2004) have not been taken into account in the estimates presented here (Tables 1 and 2) because this would have exceeded the scope of this paper owing to few estimates of bird population declines in the twenty-first century.

**Table 1:** Estimated standing biomass of the global terrestrial avifauna (expressed as fresh weight kg). Values of mean number of birds ha^−1^ (*D*) in the various biome classes taken from Gaston et al. (2003), areas of the various biome classes (*Y*) based on Saugier et al. (2001). Assuming that ≈ 90% of the terrestrial bird individuals in the temperate, boreal, and arctic zones and ≈ 60% in the tropics are arthropod-eaters (see Assumption 1, “Methods” section), it is deduced that the biomass of the world’s insectivorous birds might be ≈ 3 million tons

Biome class	Mean density (birds ha^−1^) (*D*)	Mean body weight (kg bird^−1^) (*W*)	Area (ha) (*Y*)	Biomass across biome (kg) (*D*) × (*W*) × (*Y*)
Tropical forests	20.00	0.0320^a^	1750 × 10^6^	1120 × 10^6^
Temperate and boreal forests	10.00	0.0270^b^	2410 × 10^6^	651 × 10^6^
Tropical grasslands and savannas/Mediterranean shrubland	9.25	0.0340^c^	3040 × 10^6^	956 × 10^6^
Temperate grasslands	4.00	0.0450^d^	1500 × 10^6^	270 × 10^6^
Cropland	3.00	0.0380^e^	1350 × 10^6^	154 × 10^6^
Deserts	1.75	0.1558^f^	2770 × 10^6^	755 × 10^6^
Arctic tundra	2.00	0.0674^g^	560 × 10^6^	75 × 10^6^
Global total (without ice-covered area)	–	–	13,380 × 10^6^	3981 × 10^6^

^a^Terborgh et al. 1990

^b^Holmes and Sturges 1975; Wiens and Nussbaum 1975

^c^Howell 1971; Karr 1971

^d^Wiens 1973

^e^Kartanas 1989

^f^Gutzwiller and Barrow 2002

^g^Sokolov et al. 2012; Del Hoyo et al. 2016

**Table 2:** Estimated annual consumption of arthropod prey (fresh weight) of the global population of insectivorous birds. Values for temperate biomes refer to residents and breeding migrants combined; values for tropical biomes refer to residents and non-breeding migrants combined. Not included in these calculations are the amounts of arthropod prey consumed at migration stopover sites

Biome class	Number of assessments	Prey consumption (kg ha^−1^ year^−1^)^*^	Area (ha)	Prey consumption of entire area (kg year^−1^)
		(*X*)	(*Y*)	(*X*) × (*Y*)
Tropical forests^a^	7	112.5 ± 9.2	1750 × 10^6^	196,875 × 10^6^
Temperate and boreal forests^b^	44	44.1 ± 6.2	2410 × 10^6^	106,281 × 10^6^
Tropical grasslands and savannas/Mediterranean shrubland^c^	7	15.8 ± 2.8	3040 × 10^6^	48,032 × 10^6^
Temperate grasslands (incl. meadows, pastures, old fields)^d^	11	7.5 ± 0.9	1500 × 10^6^	11,250 × 10^6^
Cropland^e^	8	20.9 ± 9.0	1350 × 10^6^	28,215 × 10^6^
Deserts^f^	18	4.1 ± 0.8	2770 × 10^6^	11,357 × 10^6^
Arctic tundra^g^	8	4.6 ± 1.3	560 × 10^6^	2576 × 10^6^
Global total (without ice-covered area)	103	–	13,380 × 10^6^	404,586 × 10^6^

^a^Karr (1975); Leigh and Smythe (1978); Reagan and Waide (1996); Robinson et al. (2000); Sakai (2002)

^b^Tima (1957); Uramoto (1961); West and DeWolfe (1974); Holmes and Sturges (1975); Karr (1975); Alatalo (1978); Szaro and Balda (1979); Smith and MacMahon (1981); Wiens (1989) (modified data from Wiens and Nussbaum 1975); Wiens (1989) (modified data from Weiner and Głowaciński 1975; Głowaciński and Weiner 1980, 1983); Weathers (1983); Keast et al. (1985); Solonen (1986); Kartanas (1989); Harris (1991)

^c^Karr (1971); UNESCO (1979); Gillon et al. (1983)

^d^Diehl (1971); Wiens (1977); Rotenberry (1980b); Smith and MacMahon (1981); Głowaciński et al. (1984); combined data Faanes (1982)/Kirk et al. (1996)

^e^Wiens and Dyer (1975); Woronecki and Dolbeer (1980); Kartanas (1989); Ferger et al. (2013)

^f^Combined data Lasiewski and Dawson (1967)/Austin (1970)

^g^Wielgolaski (1975); combined data Lasiewski and Dawson (1967)/Watson (1963); James and Rathbun (1981); Montgomerie et al. (1983); Sokolov et al. (2012)

^*^Values of prey kill (kg ha^−1^ year^−1^) presented as x̅ ± SE

### Statistical analysis of annual prey consumption in the various biomes

To determine whether prey consumption rates (kg arthropods ha^−1^ year^−1^) differed among biomes, we first determined that the consumption data among biomes were not normally distributed using normal probability plots. Rather than using a normalizing transformation, we instead performed a Kruskal-Wallis one-way analysis of variance by ranks test. The omnibus test was followed with a pairwise multiple comparison using Dunn’s test for multiple comparisons of independent samples corrected for ties (Pohlert 2018). Analyses were performed with R, the programming language (R Core Team 2018).

## Results

### Standing biomass of the global population of insectivorous birds

Based on estimates of avian standing biomass in various terrestrial biomes, we estimate the total standing biomass of the global terrestrial avifauna to be 3981 × 10^6^ kg fresh weight (= roughly 4 million metric tons; Table 1). This value is similar to an estimate of 5 million tons for the global terrestrial avifauna calculated using a different approach by Alerstam (1993). Because it is assumed that ≈ 90% of all land bird individuals in the temperate, boreal, and arctic zones and ≈ 60% in the tropics are insectivorous foragers (see Assumption 1, “Methods” section), it follows that the standing biomass of the global community of insectivorous birds might be on the order of ≈ 3 million tons (Table 1). This value is a small fraction of the global standing biomass of other predaceous animal taxa such as spiders (≈ 25 million tons; Nyffeler and Birkhofer 2017), ants (≈ 280 million tons; Hölldobler and Wilson 1994), or whales (16–103 million tons; Pershing et al. 2010). The comparatively low value of the global standing biomass of wild birds is partially explained by the fact that birds have a very low production efficiency (i.e., low P/A-ratio). With other words, in birds, the vast majority of the assimilated energy is lost in respiration and only ≈ 1–2% is converted to biomass (see Golley 1968; Holmes and Sturges 1975; Humphreys 1979).

### Prey consumption rates of insectivorous birds in the various biomes

**Fig. 1:**
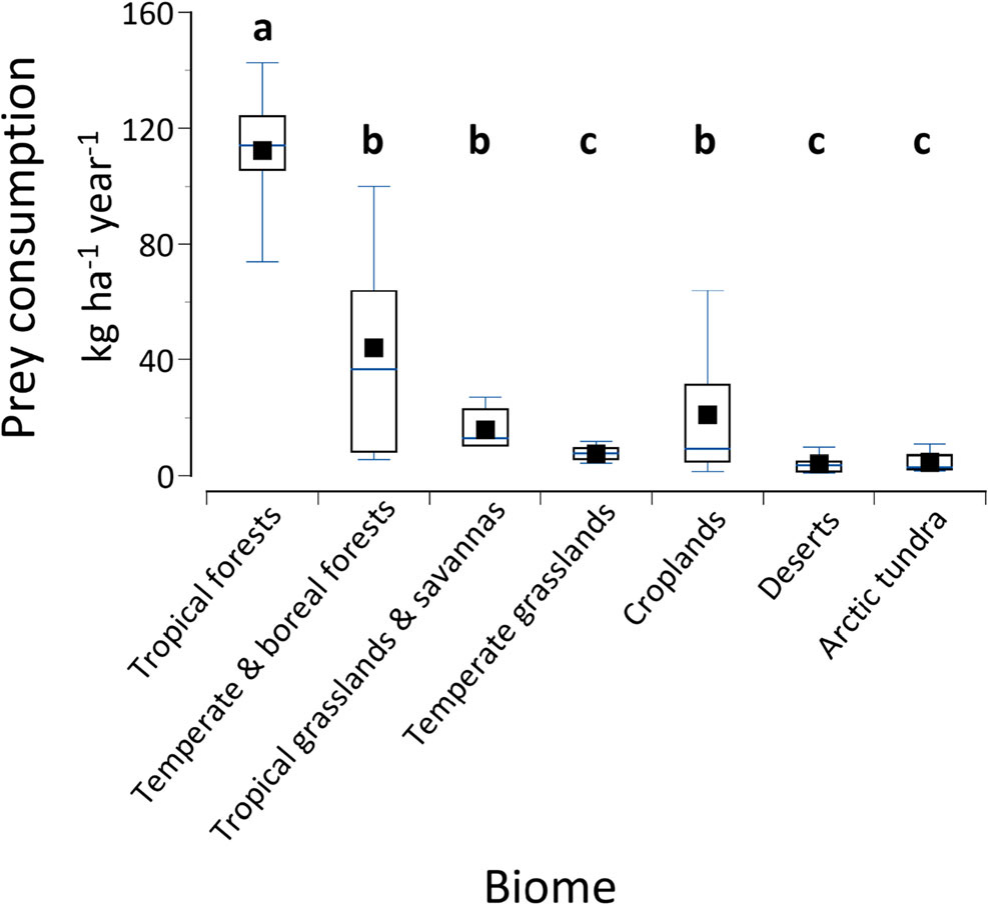
Box plots showing prey consumption rates (kg arthropods ha^−1^ year^−1^) in the various biomes. Different small case letters above boxes indicate significant differences (Kruskal-Wallis test followed by Dunn’s multiple comparison test; see text for details). High and low whiskers indicate 90th and 10th percentiles, respectively. Tops and bottoms of the boxes indicate 75th and 25th percentiles, respectively. The horizontal bars within the boxes indicate the median, and the symbols within the boxes indicate the mean biomass consumed

Prey consumption rates (kg arthropods ha^−1^ year^−1^) varied significantly among biomes (Kruskal-Wallis chi-squared = 51.179, df = 6, *P* < 0.001; Fig. 1). A Dunn’s post hoc multiple comparison test revealed that prey consumption in tropical forests was greater than in all other biomes (all *P* ≤ 0.022). Prey consumption in temperate-boreal forests was greater than in tundra (*P* < 0.001), desert (*P* < 0.001), and temperate grasslands (*P* = 0.009), but did not differ from tropical grasslands and croplands. Prey consumption was greater in tropical grassland and savanna than in desert (*P* = 0.004) and tundra (*P* = 0.024). Finally, prey consumption was greater in cropland than in desert (*P* = 0.044). Prey consumption did not differ significantly among any of the remaining biomes. Annual prey consumption correlated positively with net primary production among biomes, using NPP values from Chapin et al. (2011).

### Estimate of the global annual prey consumption by the insectivorous birds

Our calculation of the annual prey consumption by the global population of insectivorous birds produced an estimate of 404,586 × 10^6^ kg year^−1^ (= 404.6 million tons; Table 2), which corresponds to an energy consumption of ≈ 2.7 × 10^18^ J year^−1^ (= 0.15% of the global terrestrial net primary production of 1.782 × 10^21^ J year^−1^ (see Lieth 1973)).

This estimate (Table 2) does not include the amounts of food consumed at stopover sites during the fall and spring migrations. Currently, no quantitative assessments of the prey biomass consumed at stopover sites have been published (also see Lott et al. 2006); but considering the energy costs of approximately 10 to 20 billion birds migrating annually (see Hahn et al. 2009; Berthold 2001; Wikelski et al. 2003; Fristoe 2015) and taking into account that the birds resting at stopover sites only partially depend on arthropod food (Schaub and Jenni 2000; Suthers et al. 2000), we estimate that the amount of arthropod food they consume globally at stopover sites may be on the order of 3–5 million tons year^−1^. Thus, arthropod consumption during migratory stopovers is around 1% of the total amount of prey biomass consumed by the global population of insectivorous birds (see Table 2).

Regarding the temperate, sub-polar, and polar climates, our calculations (Table 2) assume that the arthropod consumption in these climates during the non-breeding season is reduced to a small fraction (≈ 5–10%) of the breeding season value (see “Methods”). However, there are some studies which indicate that the insectivorous activities of birds during the non-breeding season may not always be reduced so drastically—at least in some parts of the temperate/cold climate zones (see Askenmo et al. 1977; Gunnarsson 1996; Kirk et al. 1996; Vel’ký et al. 2011) —and it could therefore be argued our calculations underestimate the contribution of birds as consumers of arthropod prey during the non-breeding period (Table 2). To address this issue, we considered two extreme scenarios. In scenario 1, a minimum estimate was assessed based on the assumption that the birds’ diets in temperate/cold climates contain no arthropods during the non-breeding season; in scenario 2, a maximum estimate was assessed by assuming that the birds’ contribution as arthropod consumers during the non-breeding season in temperate/cold climates is 50% of the breeding season value. With these assumptions, the annual prey consumption of the world’s insectivorous birds was recalculated, producing a minimum estimate of 396,041 million tons year^−1^ and a maximum estimate of 472,145 million tons year^−1^. Thus, the true value of insect consumption presumably is somewhere in between approximately 400 and 500 million tons year^−1^, but most likely at the lower end of this range as indicated in Table 2, because the availability of arthropod prey during the non-breeding season is greatly reduced in most areas of the temperate/cold climates.

For comparison, Alerstam (1993), using a different method, estimated the total energy consumption of the world’s land birds (including arthropods, plant matter, and other food sources) to be ≈ 7.5 × 10^18^ J year^−1^. Our estimate for the world’s insectivorous birds is consistent with this broader estimate. The difference of 4.6 × 10^18^ J year^−1^ between the two estimates is mainly explained by the fact that in our estimate exclusively feeding on arthropod prey is considered, whereas in Alerstam’s estimate, feeding on additional food sources was assumed. Especially during the non-breeding season, when the availability of arthropod prey is strongly reduced in many places of the globe, land birds consume large amounts of plant matter (Clements and Shelford 1939; Brown et al. 1979; Robinson and Sutherland 1997; Buckingham et al. 1999; Renner et al. 2012).

## Discussion

### Experimental evidence supporting our theory of high global predation impact by insectivorous birds

Our calculations presented in Table 2 imply that insectivorous birds exert substantial predation pressure on insects and other arthropods, especially in tropical and temperate/boreal forest ecosystems. This is supported by a large number of experimental studies conducted in a variety of habitats in different parts of the world (see Şekercioğlu 2006a, Mäntylä et al. 2011; Şekercioğlu et al. 2016 for reviews). Thereby, exclosure experiments were used to document the impact of bird predation on arthropods (Whelan et al. 2008). With this technique, it has been proven that birds can significantly reduce the abundance of herbivorous insects in tropical, temperate, and boreal forests (Holmes et al. 1979; Gradwohl and Greenberg 1982; Atlegrim 1989; Marquis and Whelan 1994; Gunnarsson 1996; Murakami and Nakano 2000; Strong et al. 2000; Van Bael et al. 2003; Dunham 2008; Morrison and Lindell 2012). Exclosure experiments also show that insectivorous birds can also have a negative effect on the abundance of herbivorous insects in grasslands (Joern 1986; Bock et al. 1992) and croplands (Hooks et al. 2003; Perfecto et al. 2004; Kellermann et al. 2008; Koh 2008; Johnson et al. 2010; Maas et al. 2016).

Negative effects of insectivorous birds on herbivorous insects have been further demonstrated by means of dummy caterpillar experiments in tropical and non-tropical biomes (e.g., Maas et al. 2015; Roslin et al. 2017).

### Which prey taxa are killed by insectivorous birds?

Insectivorous birds eat a large variety of arthropod taxa (e.g., Rotenberry 1980b; Poulin et al. 1994; Dyrcz and Flinks 1995; Gajdoš and Krištín 1997; Orłowski et al. 2014; Helms et al. 2016; Sam et al. 2017). Seven arthropod orders, Lepidoptera, Coleoptera, Orthoptera, Diptera, Hemiptera, Hymenoptera, and Araneae, however, are frequently consumed (Gajdoš and Krištín 1997; Wilson et al. 1999; Develey and Peres 2000; Gámez-Virués et al. 2007; Sam et al. 2017). In temperate forests and agricultural habitats, caterpillars (Lepidoptera larvae) and beetles (Coleoptera) are particularly common prey of insectivorous birds (Holmes et al. 1979; Woronecki and Dolbeer 1980; Gajdoš and Krištín 1997; Jeromin 2002; Fayt et al. 2005; Moorman et al. 2007; Gilroy et al. 2009; Pagani-Núñez et al. 2017), whereas grasshoppers (Orthoptera) are usually an essential component in the diets of grassland birds (Joern 1986; Bock et al. 1992; Kobal et al. 1998). Tropical forest and farmland birds frequently consume beetles, ants, cockroaches (Blattodea), katydids (Orthoptera), caterpillars, and spiders (Poulin and Lefebvre 1996; Şekercioğlu et al. 2002; Hooks et al. 2003; Koh 2008; Sam et al. 2017). Desert birds frequently feed on beetles, ants, and termites (Maclean 2013). Termites are an important food source for birds inhabiting tropical savannas (Korb and Salewski 2000). In Arctic tundra habitats, birds consume mostly tipulids (Diptera) and spiders (Araneae)—two arthropod groups numerically dominating the arthropod fauna of the sparse tundra vegetation (Holmes 1966; Custer and Pitelka 1978).

### Relative contribution of different biome categories to the global annual prey consumption

Birds in forests account for 75% of the annual prey consumption of the world’s insectivorous birds (≈ 300 million tons year^−1^; Table 2). Forests cover a large portion of the global terrestrial surface area (41.6 million km^2^; Saugier et al. 2001), and in these productive and vegetatively complex habitats, birds usually reach higher diversities (Willson 1974) and numbers ha^−1^ compared to non-forested areas (Gaston et al. 2003). A similar trend of highest predation impact occurring in forested areas has been reported for spiders (Nyffeler and Birkhofer 2017). Forest birds feed frequently on potentially harmful caterpillar and beetle pests (Holmes et al. 1979; Fayt et al. 2005; Moorman et al. 2007). This is especially true during the breeding season, when passerines (song birds) catch large numbers of leaf-eating caterpillars to feed them to their nestlings (Gibb and Betts 1963; Holmes et al. 1979; Gajdoš and Krištín 1997; Mols and Visser 2002). At this time of the year, caterpillars make up 20–90% of the nestling diets of many species of insectivorous birds (Gibb and Betts 1963; Pravosudov and Pravosudova 1996; Gajdoš and Krištín 1997; Török and Tóth 1999; Pagani-Núñez et al. 2017). Due to high protein content and easy digestibility, caterpillars comprise an optimal diet for nestling birds (Tremblay et al. 2005). Data suggest that forest birds exert considerable predation pressure on lepidopteran pests, such as the eastern spruce budworm (*Choristoneura fumiferana*; Holmes et al. 1979; Şekercioğlu 2006a). Crawford and Jennings (1989) found that birds destroyed 84% of larval and pupal eastern spruce budworms at low densities of this pest. The birds are most effective as natural enemies at endemic pest densities (Holmes et al. 1979; Holmes 1990). Fayt et al. (2005) pointed out that woodpeckers (Picidae) suppress the abundance of bark beetles (Curculionidae) in coniferous forest landscapes. Furthermore, forest birds at times feed heavily on spiders, especially during the breeding season (Naef-Daenzer et al. 2000; Pagani-Núñez et al. 2017). In Scandinavian boreal forests, spiders are a major diet for overwintering tits (*Parus* spp.), treecreepers (*Certhia familiaris*), and goldcrests (*Regulus regulus*) (Askenmo et al. 1977; Gunnarsson 1996). Spiders are an important food source for birds because of their high content of taurine, an amino acid that plays a vital role in the early development of many types of passerine birds (Ramsay and Houston 2003; Arnold et al. 2007). The propensity for birds to feed on spiders can reduce some positive economic impact of avian insectivory because spiders themselves are highly beneficial natural enemies of insects (Nyffeler 2000; Nyffeler and Birkhofer 2017). The same is true when birds feed on large numbers of predaceous ants or odonates, as is sometimes the case in purple martins (*Progne subis*) and house martins (*Delichon urbicum*) (Kelly et al. 2013; Orłowski et al. 2014; Helms et al. 2016).

Birds in grasslands and savannas contributed 15% (i.e., ≈ 60 million tons year^−1^; Table 2) to the global annual prey biomass. Grasslands and savannas cover a vast area of the globe (45.4 million km^2^; Saugier et al. 2001). Included in this figure are 2.8 million km^2^ Mediterranean shrublands. The prey biomass ha^−1^ year^−1^ of bird communities in the grassland biome is considerably lower than that in forests (Table 2; Ford and Bell 1981; Wiens 1989). Notwithstanding that, North American studies have shown that grassland birds at times exert noticeable predation pressure on grasshopper populations (Joern 1986; Belovsky et al. 1990; Bock et al. 1992).

Bird communities associated with agricultural areas contributed roughly 7% (i.e., ≈ 28 million tons year^−1^; Table 2). Cropland covers an area of 13.5 million km^2^ (Saugier et al., 2001) Agricultural landscapes are mosaics of crop fields, shelterbelts, and tree-lined field roads (Kartanas 1989; Gámez-Virués et al. 2007). In our estimates of prey biomass ha^−1^ year^−1^ for croplands (Table 2), birds associated with tree-lined field roads have been taken into account as well (compare Kartanas 1989). Although birds in the agricultural landscape are known to feed at times heavily on potentially harmful lepidopteran and coleopteran pests (Woronecki and Dolbeer 1980), examples of farmland birds substantially suppressing crop pests are few, which may be explained by the fact that crop fields are usually inhabited/visited by birds in rather low numbers (Gaston et al. 2003), at least in temperate regions. Reports of birds suppressing agricultural pests refer for the most part to studies in tropical plantations (e.g., Hooks et al. 2003; Koh 2008). A classic example of the successful avian control of a pest species comes from tropical coffee plantations in Costa Rica, Guatemala, Jamaica, Mexico, Panama, and Puerto Rico, where the coffee berry borer *Hypothenemus hampei*—considered to be the world’s most damaging insect pest in coffee—is successfully controlled by insectivorous avian communities often largely composed of wood-warblers (Parulidae) (Greenberg et al. 2000; Perfecto et al. 2004; Kellermann et al. 2008; Johnson et al. 2010; Wenny et al. 2011; Karp et al. 2013).

Birds associated with desert and tundra biomes account for only a small percentage (each < 4%) of the global annual prey biomass (Table 2). The low prey biomass ha^−1^ year^−1^ of birds in these biome types reflects that such habitats are covered by a sparse vegetation of low productivity supporting only low densities of birds (see Gaston et al. 2003). Birds in desert and tundra habitats prey exclusively on non-pest arthropods during their occurrence in these biomes which renders them insignificant from the perspective of economic ornithology (Holmes 1966; Custer and Pitelka 1978; Maclean 2013).

## Concluding remarks

For the first time, the predation impact of the insectivorous birds has been quantified on a global scale. The global energy consumption by the insectivorous birds in the form of arthropod prey is substantial (an estimated ≈ 2.7 × 10^18^ J year^−1^). Annually, the global population of insectivorous birds consumes as much energy as a megacity the size of New York (≈ 2.8 × 10^18^ J year^−1^, in 2011; Kennedy et al. 2015).

To fulfill these huge energy requirements, the insectivorous birds capture billions of potentially harmful herbivorous insects and other arthropods. Only few other predator groups, such as spiders and entomophagous insects, can keep up with the insectivorous birds in their capacity to suppress herbivorous insect populations in a variety of biomes (Table 3; DeBach and Rosen 1991; Nyffeler and Birkhofer 2017). Other predator groups like bats, primates, shrews, hedgehogs, frogs, salamanders, and lizards apparently are less effective natural enemies of herbivorous insects (Table 3). Although some of these latter predator groups may exert high predation pressure in a particular biome type (e.g., lizards on tropical islands; see Bennett and Gorman 1979), these same groups are much less effective in other biomes so that their global impact cannot compare to that of spiders, entomophagous insects, or insectivorous birds. The global predation impact of the insectivorous birds (between 400 and 500 million tons year^−1^) is approximately of the same order of magnitude as that of the spiders (between 400 and 800 million tons year^−1^; see Nyffeler and Birkhofer 2017).

**Table 3:** Comparative estimates of the annual prey consumption (kg fresh weight ha^−1^ year^−1^) of different groups of predaceous animals based on published data

Predator type	Biome class	Prey biomass^a^ (kg ha^−1^ year^−1^)	Source
Vertebrates:			
Insectivorous birds	Salt marsh	545	Kale 1965
Insectivorous birds	Urban areas	84–289	Falk 1976; Kartanas 1989
Insectivorous birds	Tropical forests	100–176	Leigh 1999
Insectivorous birds	Temperate forests	35–137	Holmes and Sturges 1975; Weiner and Głowaciński 1975; Keast et al. 1985; Harris 1991
Insectivorous birds	Tree-lined field roads	36–79	Kartanas 1989
Insectivorous birds	Grasslands, crop fields	10–31	Ferger et al. 2013; Wiens and Dyer 1975
Piscivorous birds	Freshwater lakes and marshes	8–49	Nilsson and Nilsson 1976; Biujse et al. 1993
Insectivorous primates	Tropical forest	10–32	Sakai (2002)
Insectivorous bats	Tropical forest	4	Kalka and Kalko 2006
Insectivorous bats	Carlsbad Caverns national park	Low^b^	Combined data Tuttle 1994/ Best and Geluso 2003
Shrews	Taiga forest	25–350	Shvarts et al. 1997
Shrews	Reed swamp	6	Pelikan 1978
Hedgehogs	Reed swamp	1	Pelikan 1978
Lizards	Various biome types	3–9	Shelly 1986; Walter and Breckle 2013
Lizards	Woodland on tropical island	85	Bennett and Gorman 1979
Salamanders	Temperate forests	7	Burton and Likens 1975
Frogs	Tropical forest	1–163	Stewart and Woolbright 1996; Walter and Breckle 2013
Frogs	Temperate grasslands	< 1–180	Breymeyer 1978; Pelikan 1993
Invertebrates:			
Ants	Tropical forest	21–147	Dyer 2002
Ants	Temperate forest	177	Horstmann 1974
Ants	Temperate grasslands	46–536	Kajak et al. 1971
Spiders	Tropical coffee plantation	160–320	Robinson and Robinson 1974
Spiders	Temperate forests	20–100	Nyffeler 2000; Nyffeler and Birkhofer 2017
Spiders	Temperate grasslands	20–230	Nyffeler 2000; Nyffeler and Birkhofer 2017
Spiders	Crop fields	≤ 10	Nyffeler 2000
Scorpions	Arid zone	8	Shorthouse and Marples 1982
Wasps (Vespa)	Temperate forest	1–8	Harris 1991
Robber flies (Asilidae)	Tropical forest	7	Shelly 1986
Ground beetles (Carabidae)	Temperate forest, cropland	20	Chauvin 1967; Schaefer 1990
Rove beetles (Staphylinidae)	Temperate forest	64	Schaefer 1990
Centipedes	Temperate forest	100	Schaefer 1990

^a^Original values adjusted when necessary by using correction factors obtained from the literature

^b^Only a few kg arthropods ha^−1^ year^−1^ (Nyffeler, unpubl. estimate), taking into account a foraging area with a radius of ≈ 50 km for the Mexican free-tailed bat (Best and Geluso 2003)

## Electronic supplementary material

ESM 1 (DOCX 122 kb)
