# Supplementary material for: Insectivorous birds consume an estimated 400–500 million tons of prey annually
Source: Naturwissenschaften. 2018 Jul 9;105(7):47. doi: 10.1007/s00114-018-1571-z (PMC6061143; doi:10.1007/s00114-018-1571-z)
Supplement: Supplementary file 1 — (DOCX 122 kb) [file 114_2018_1571_MOESM1_ESM.docx]

Supplementary Material: Insectivorous birds consume an estimated 400-500 million tons of prey annually – M Nyffeler, ÇH Şekercioğlu & CJ Whelan

| **Record #** | **Biome type** | **Location / Literature source** | **Original value published in the literature** | **kg fw arthropods killed ha^-1^ season^-1^**  Breeding period  (BP) | **kg fw arthropods killed ha^-1^ season^-1^**  Nonbreeding period ^A^  (NP) | **kg fw arthropods killed ha^-1^ year^-1^**  (BP) + (NP) |
| --- | --- | --- | --- | --- | --- | --- |
| 1 | Tropical forest | Cocha Cashu, Peru / Robinson et al. 2000 | 20 kg dw arthropods consumed ha^-1^ yr^-1^ | 66.6 |  | 66.6 |
| 2 | Tropical forest | Limbo, Panama / Robinson et al. 2000 | 31 kg dw arthropods consumed ha^-1^ yr^-1^ | 103.2 |  | 103.2 |
| 3 | Most tropical forests | Mainland Panama / Leigh & Smythe 1978 | 34 kg dw arthropods consumed ha^-1^ yr^-1^ | 113.2 |  | 113.2 |
| 4 | Moist tropical forest | Mt. Nimba, Liberia / Leigh & Smythe 1978 | 38 kg dw arthropods consumed ha^-1^ yr^-1^ | 126.5 |  | 126.5 |
| 5 | Tropical island forest | Puercos Island, Panama / combined data from Karr 1975; Leigh & Smythe 1978 | 35.16 kg dw arthropods consumed ha^-1^ yr^-1^ | 117.1 |  | 117.1 |
| 6 | Tropical forest | Samangoh, Malaysia / Sakai 2002 | 44 kg dw arthropods consumed ha^-1^ yr^-1^ | 146.5 |  | 146.5 |
| 7 | Tropical forest | Puerto Rico / Waide 1996 | Energy flow through the bird community 1289 MJ ha^-1^ yr^-1^ (=energy assimilated) | 114.2 ^B^ |  | 114.2 |
| 8 | Pine forests | Latvian Soviet Socialist Republic / Tima 1957 | 18,000 kg fw arthropods (200 ha)^-1^ yr^-1^ | 90.0 |  | 90.0 |
| 9 | Broad-leaved deciduous forest | Japan / Uramoto 1961 | 0.3855 kg fw food ha^-1^ day^-1^ x 150 days breeding season | 39.0 | 2.9 | 41.9 |
| 10 | Northeastern deciduous forest | New Hampshire, USA / Holmes & Sturges 1975 [modified by Wiens 1989] | Energy flow (consumption) through bird community 413.3 MJ ha^-1^ yr^-1^ | 41.3 |  | 41.3 |
| 11 | Temperate deciduous forest | Illinois, USA / Karr 1975 | Annual energy requirements of avifauna 435 MJ ha^-1^ yr^-1^ (= energy assimilated) ^C^ | 115.60 |  | 115.6 |
| 12 | Pine forest | Finland / Alatalo 1978 | Energy flow (consumption) through bird community 71 MJ ha^-1^ yr^-1^ | 5.57 ^D^ |  | 5.57 |
| 13 | Spruce forest | Finland / Alatalo 1978 | Energy flow (consumption) through bird community 99 MJ ha^-1^ yr^-1^ | 7.78 ^D^ |  | 7.78 |
| 14 | Mixed coniferous forest | Finland / Alatalo 1978 | Energy flow (consumption) through bird community 119 MJ ha^-1^ yr^-1^ | 9.36 ^D^ |  | 9.36 |
| 15 | Spruce-deciduous forest | Finland / Alatalo 1978 | Energy flow (consumption) through bird community 124 MJ ha^-1^ yr^-1^ | 9.74 ^D^ |  | 9.74 |
| 16 | Mixed forest | Finland / Alatalo 1978 | Energy flow (consumption) through bird community 149 MJ ha^-1^ yr^-1^ | 11.70 ^D^ |  | 11.70 |
| 17 | Afforested swamp | Finland / Alatalo 1978 | Energy flow (consumption) through bird community 107 MJ ha^-1^ yr^-1^ | 8.42 ^D^ |  | 8.42 |
| 18 | Young forest: | Finland / Alatalo 1978 | Energy flow (consumption) through bird community 86 MJ ha^-1^ yr^-1^ | 6.75 ^D^ |  | 6.75 |
| 19 | Aspen forests | Utah, USA / Smith & MacMahon 1981 | Energy flow (consumption) through bird community 173.5 MJ ha^-1^ yr^-1^ | 17.33 |  | 17.33 |
| 20 | Fir forests | Utah, USA / Smith & MacMahon 1981 | Energy flow (consumption) through bird community 335.0 MJ ha^-1^ yr^-1^ | 33.47 |  | 33.47 |
| 21 | Spruce forests | Utah, USA / Smith & MacMahon 1981 | Energy flow (consumption) through bird community 301.5 MJ ha^-1^ yr^-1^ | 30.12 |  | 30.12 |
| 22 | Managed pine forest (clear cut) | Arizona, USA / Szaro & Balda 1979 [modified by Wiens 1989] | Energy flow (consumption) through bird community 8.1-12.8 MJ ha^-1^ breeding season^-1^  → x̅ = 10.45 MJ ha^-1^ breeding season^-1^ | 1.03 | 0.08 | 1.11 |
| 23 | Managed pine forest (selectively thinned) | Arizona, USA / Szaro & Balda 1979 [modified by Wiens 1989] | Energy flow (consumption) through bird community 39.1-63.2 MJ ha^-1^ breeding season^-1^ → x̅ = 51.15 MJ ha^-1^ breeding season^-1^ | 5.10 | 0.38 | 5.48 |
| 24 | Managed pine forest (strip cut) | Arizona, USA / Szaro & Balda 1979 [modified by Wiens 1989] | Energy flow (consumption) through bird community 45.1-96.4 MJ ha^-1^ breeding season^-1^ → x̅ = 70.75 MJ ha^-1^ breeding season^-1^ | 7.06 | 0.53 | 7.59 |
| 25 | Managed pine forest (silviculturally cut) | Arizona, USA / Szaro & Balda 1979 [modified by Wiens 1989] | Energy flow (consumption) through bird community 42.8-85.5 MJ ha^-1^ breeding season^-1^ → x̅ = 64.15 MJ ha^-1^ breeding season^-1^ | 6.41 | 0.48 | 6.89 |
| 26 | Managed pine forest (uncut control) | Arizona, USA / Szaro & Balda 1979 [modified by Wiens 1989] | Energy flow (consumption) through bird community 34.0-78.9 MJ ha^-1^ breeding season^-1^ → x̅ = 56.45 MJ ha^-1^ breeding season^-1^ | 5.64 | 0.42 | 6.06 |
| 27 | Low-elevation dry douglas-fir forest | Oregon, USA / Wiens 1989 | Energy flow (consumption) through bird community 499.6 MJ ha^-1^ breeding season^-1^ | 49.90 | 3.74 | 53.64 |
| 28 | Low-elevation mesic hemlock forest | Oregon, USA / Wiens 1989 | Energy flow (consumption) through bird community 439.5 MJ ha^-1^ breeding season^-1^ | 43.90 | 3.29 | 47.19 |
| 29 | Low-elevation moist hemlock forest | Oregon, USA / Wiens 1989 | Energy flow (consumption) through bird community 697.6 MJ ha^-1^ breeding season^-1^ | 69.68 | 5.23 | 74.91 |
| 30 | Mid-elevation transitional forest | Oregon, USA / Wiens 1989 | Energy flow (consumption) through bird community 869.8 MJ ha^-1^ breeding season^-1^ | 86.90 | 6.52 | 93.42 |
| 31 | High-elevation dry fir hemlock forest | Oregon, USA / Wiens 1989 | Energy flow (consumption) through bird community 509.1 MJ ha^-1^ breeding season^-1^ | 50.87 | 3.82 | 54.69 |
| 32 | High-elevation moist fir forest | Oregon, USA / Wiens 1989 | Energy flow (consumption) through bird community 513.7 MJ ha^-1^ breeding season^-1^ | 51.31 | 3.85 | 55.16 |
| 33 | Deciduous forests (pioneer stage) | Poland / Glowacinski & Weiner 1983 [modified by Wiens 1989] | Energy flow (consumption) through bird community 58.7 MJ ha^-1^ breeding season^-1^ | 5.87 | 0.44 | 6.31 |

| 34 | | Deciduous forests (15-yr) | Poland / Glowacinski & Weiner 1983 [modified by Wiens 1989] | Energy flow (consumption) through bird community 925.1 MJ ha^-1^ breeding season^-1^ | 92.43 | 6.93 | 99.36 |
| --- | --- | --- | --- | --- | --- | --- | --- |
| 35 | | Deciduous forests (20-30 yr) | Poland / Glowacinski & Weiner 1983 [modified by Wiens 1989] | Energy flow (consumption) through bird community 536.6 MJ ha^-1^ breeding season^-1^ | 53.61 | 4.02 | 57.63 |
| 36 | | Deciduous forests (climax) | Poland / Glowacinski & Weiner 1983 [modified by Wiens 1989] | Energy flow (consumption) through bird community 981.0 MJ ha^-1^ breeding season^-1^ | 98.00 | 7.35 | 105.35 |
| 37 | | Deciduous forests (mixed alder-oak) | Poland / Glowacinski & Weiner 1983 [modified by Wiens 1989] | Energy flow (consumption) through bird community 1136.0 MJ ha^-1^ breeding season^-1^ | 113.49 | 8.51 | 122.00 |
| 38 | | Deciduous forests (oak-hornbeam) | Poland / Weiner & Glowacinski 1975 [modified by Wiens 1989] | Energy flow (consumption) through bird community 632.4 MJ ha^-1^ breeding season^-1^ | 63.19 | 4.74 | 67.93 |
| 39 | | Managed pine forests (1-yr clear-cut) | Poland / Weiner & Glowacinski 1980 [modified by Wiens 1989] | Energy flow (consumption) through bird community 15.2 MJ ha^-1^ breeding season^-1^ | 1.53 | 0.11 | 1.64 |
| 40 | | Managed pine forests (4-yr culture) | Poland / Glowacinski & Weiner 1980 [modified by Wiens 1989] | Energy flow (consumption) through bird community 98.8 MJ ha^-1^ breeding season^-1^ | 9.87 | 0.74 | 10.61 |
| 41 | | Managed pine forests (10-yr thicket) | Poland / Glowacinski & Weiner 1980 [modified by Wiens 1989] | Energy flow (consumption) through bird community 292.2 MJ ha^-1^ breeding season^-1^ | 29.20 | 2.19 | 31.39 |
| 42 | | Managed pine forests (35-yr pole pine) | Poland / Glowacinski & Weiner 1980 [modified by Wiens 1989] | Energy flow (consumption) through bird community 285.0 MJ ha^-1^ breeding season^-1^ | 28.48 | 2.14 | 30.62 |
| 43 | | Managed pine forests (80-yr forest) | Poland / Glowacinski & Weiner 1980 [modified by Wiens 1989] | Energy flow (consumption) through bird community 568.6 MJ ha^-1^ breeding season^-1^ | 56.80 | 4.26 | 61.06 |
| 44 | | Pinon-Juniper woodland | California, USA / Weathers 1983 | Energy flow (assimilated) through bird community ≈500 MJ ha^-1^ yr^-1^ | 66.43 |  | 66.43 |
| 45 | | Coniferous forest | California, USA / Weathers 1983 | Energy flow (assimilated) through bird community ≈300 MJ ha^-1^ yr^-1^ | 39.85 |  | 39.85 |
| 46 | | Eucalypt forests | New South Wales, Australia / Keast 1985 | Energy flow (insect consumption) through bird community 552.29-778.22 MJ ha^-1^ yr^-1^ (x̅ = 665.25 MJ ha^-1^ yr^-1^) | 98.47 |  | 98.47 |
| 47 | | Luxuriant mixed forests | Finland / Solonen 1986 | Energy flow (consumption) through bird community 1872 MJ ha^-1^ yr^-1^ | 187.01 |  | 187.01 |
| 48 | | Temperate forest | Poland / Kartanas (1989) | Energy flow (consumption) through bird community 378.74 MJ ha^-1^ breeding season^-1^ | 37.83 | 2.84 | 40.67 |
| 49 | | Temperate forest | Poland / Kartanas (1989) | Energy flow (consumption) through bird community 551.26 MJ ha^-1^ breeding season^-1^ | 55.07 | 4.13 | 59.20 |
| 50 | | Honeydew beech forest | New Zealand / Harris 1991 | Insect consumption 4.0-35.4 kg fw ha^-1^ yr^-1^  → x̅ = 19.7 kg fw ha^-1^ yr^-1^ | 19.7 |  | 19.7 |
| 51 | | Taiga forest | Alaska / West & DeWolfe 1974 | 193 kg fw (100 ha)^-1^ breeding season^-1^ | 1.39 | 0.10 | 1.49 |
| 52 | | Tropical grazing land | Ivory Coast / UNESCO (1979) | 10 kg fw arthropods ha^-1^ year^-1^ | 10.0 |  | 10.0 |
| 53 | | Tropical savannah | Senegal / Gillon et al. (1983) | 26.1 kg fw arthropods consumed ha^-1^ yr^-1^ | 26.1 |  | 26.1 |
| 54 | | Tropical savannah | Senegal / Gillon et al. (1983) | 27.2 kg fw arthropods consumed ha^-1^ yr^-1^ | 27.2 |  | 27.2 |
| 55 | | Tropical grassland (dry grazed) | Panama / Karr (1971) Field metabolism = 2.0 × EMR (existence energy) | 174 kJ day^-1^ existence energy × 365 days → 63.51 MJ year^-1^ (assimilated energy) ^C^ | 11.3 |  | 11.3 |
| 56 | | Tropical grassland (wet grazed) | Panama / Karr (1971) | 198 kJ day^-1^ existence energy × 365 days → 72.27 MJ year^-1^ (assimilated energy) ^C^ | 12.8 |  | 12.8 |
| 57 | | Tropical grassland (dry ungrazed) | Panama / Karr (1971) | 155 kJ day^-1^ existence energy × 365 days → 56.58 MJ year^-1^ (assimilated energy) ^C^ | 10.0 |  | 10.0 |
| 58 | | Tropical grassland (wet ungrazed) | Panama / Karr (1971) | 201 kJ day^-1^ existence energy × 365 days → 73.37 MJ year^-1^ (assimilated energy) ^C^ | 13.0 |  | 13.0 |
| 59 | | Ungrazed grassland | Washington, USA / Wiens (1977) | 1.66 kg dw arthropods consumed ha^-1^ breeding season^-1^ (3 yr mean) | 5.53 | 0.41 | 5.94 |
| 60 | | Grazed grassland | Texas, USA / Wiens (1977) | 2.51 kg dw arthropods consumed ha^-1^ breeding season^-1^ (3 yr mean) | 8.36 | 0.63 | 8.99 |
| 61 | | Ungrazed grassland | Texas, USA / Wiens (1977) | 1.67 kg dw arthropods consumed ha^-1^ breeding season^-1^ (3 yr mean) | 5.56 | 0.42 | 5.98 |
| 62 | | Grazed grassland | South Dakota, USA / Wiens (1977) | 1.58 kg dw arthropods consumed ha^-1^ breeding season^-1^ (3 yr mean) | 5.26 | 0.39 | 5.65 |
| 63 | | Ungrazed grassland | South Dakota, USA / Wiens (1977) | 2.65 kg dw arthropods consumed ha^-1^ breeding season^-1^ (3 yr mean) | 8.82 | 0.66 | 9.48 |
| 64 | | Grazed grassland | Oklahoma, USA / Wiens (1977) | 2.24 kg dw arthropods consumed ha^-1^ breeding season^-1^ (3 yr mean) | 7.46 | 0.56 | 8.02 |
| 65 | Sagebrush/bunchgrass shrubsteppe | | Washington State, USA / Rotenberry (1980) | Energy flow (consumption) through bird community 121.9 MJ ha^-1^ yr^-1^ | 11.14 ^E^ |  | 11.14 |
| 66 | Alpine meadow | | Utah, USA / Smith & MacMahon (1981) | Energy flow (consumption) through bird community 21.3 MJ ha^-1^ year^-1^ | 2.13 |  | 2.13 |
| 67 | Wet meadow | | Poland / Glowacinski et al. (1984) | Energy flow (consumption) through bird community 115.5 MJ ha^-1^ breeding season^-1^ | 11.53 | 0.86 | 12.39 |
| 68 | Meadow | | Poland / Diehl (1971) | 3.48 kg ha^-1^ month^-1^ (immatures) + 1.90 kg ha^-1^ month^-1^ (adults) consumed by Red-Backed Shrikes during the breeding season (Diehl 1971; Nyffeler, unpubl. data) → 90 days breeding season <http://www.vogelwarte.ch/en/birds/birds-of-switzerland/red-backed-shrike> | 5.38 | 0.40 | 5.78 |
| 69 | | Upland native prairie | North Dakota, USA / Combined data Faanes (1982)/Kirk et al. (1996) | → 1 pair of savannah sparrows consumes 5 kg arthropod prey per breeding season → breeding population = 1.4 pairs ha^-1^ | 7.00 | 0.53 | 7.53 |
| 70 | | Cropland | Kenya, Africa / Ferger et al. (2013) | 1,000 kg arthropods consumed km^-2^ year^-1^ | 10.00 |  | 10.00 |
| 71 | | Cereal fields | Ohio, USA / Wiens & Dyer (1975) | 244,437 kg dw arthropods (31,416 ha)^-1^ breeding season^-1^ consumed by red-winged blackbirds | 25.90 ^F^ | 1.94 | 27.84 |
| 72 | | Corn fields | Ohio, USA / Woronecki & Dolbeer (1980) | 53 kg fw arthropods (100 ha)^-1^ breeding season^-1^ consumed by redwings and their nestlings | 0.53 | 0.04 | 0.57 |
| 73 | | Wheat field | Poland / Kartanas (1989) | Energy flow (consumption) through bird community 40.29 MJ ha^-1^ breeding season^-1^ | 5.66 ^G^ | 0.42 | 6.08 |
| 74 | | Mosaic of different crops | Poland / Kartanas (1989) | Energy flow (consumption) through bird community 54.13 MJ ha^-1^ breeding season^-1^ | 7.62 ^G^ | 0.57 | 8.19 |
| 75 | | Barley field | Poland / Kartanas (1989) | Energy flow (consumption) through bird community 22.96 MJ ha^-1^ breeding season^-1^ | 3.23 ^G^ | 0.24 | 3.47 |
| 76 | | Field road lined with trees in the midst of an agricultural landscape | Poland / Kartanas (1989) | Energy flow (consumption) through bird community 323.53 MJ ha^-1^ breeding season^-1^ | 32.33 | 2.42 | 34.75 |
| 77 | | Field road lined with trees in the midst of an agricultural landscape | Poland / Kartanas (1989) | Energy flow (consumption) through bird community 712.78 MJ ha^-1^ breeding season^-1^ | 71.21 | 5.34 | 76.55 |
| 78 | | Desert | California, USA / Austin (1970) | 1.0 birds ha^-1^ × 3418 g fw prey bird^-1^ breeding season^-1^ | 1.743 ^H^ | 0.131 | 1.874 |
| 79 | | Desert | California, USA / Austin (1970) | 1.95 birds ha^-1^ × 3418 g fw prey bird^-1^ breeding season^-1^ | 3.399 ^H^ | 0.255 | 3.654 |
| 80 | | Desert | New Mexico, USA / Austin (1970) | 0.425 birds ha^-1^ × 3418 g fw prey bird^-1^ breeding season^-1^ | 0.741 ^H^ | 0.056 | 0.797 |
| 81 | | Desert | New Mexico, USA / Austin (1970) | 0.885 birds ha^-1^ × 3418 g fw prey bird^-1^ breeding season^-1^ | 1.543 ^H^ | 0.116 | 1.659 |
| 82 | | Desert | Texas, USA / Austin (1970) | 0.75 birds ha^-1^ × 3418 g fw prey bird^-1^ breeding season^-1^ | 1.307 ^H^ | 0.098 | 1.405 |
| 83 | | Desert | Texas, USA / Austin (1970) | 0.455 birds ha^-1^ × 3418 g fw prey bird^-1^ breeding season^-1^ | 0.793 ^H^ | 0.059 | 0.852 |
| 84 | | Desert | Arizona, USA / Austin (1970) | 1.85 birds ha^-1^ × 3418 g fw prey bird^-1^ breeding season^-1^ | 3.225 ^H^ | 0.242 | 3.467 |
| 85 | | Desert | Nevada, USA / Austin (1970) | 0.3 birds ha^-1^ × 3418 g fw prey bird^-1^ breeding season^-1^ | 0.523 ^H^ | 0.039 | 0.562 |
| 86 | | Desert | Nevada, USA / Austin (1970) | 0.555 birds ha^-1^ × 3418 g fw prey bird^-1^ breeding season^-1^ | 0.967 ^H^ | 0.073 | 1.040 |
| 87 | | Desert | Nevada, USA / Austin (1970) | 2.21 birds ha^-1^ × 3418 g fw prey bird^-1^ breeding season^-1^ | 3.852 ^H^ | 0.289 | 4.141 |
| 88 | | Desert | Nevada, USA / Austin (1970) | 2.44 birds ha^-1^ × 3418 g fw prey bird^-1^ breeding season^-1^ | 4.253 ^H^ | 0.319 | 4.572 |
| 89 | | Desert | Texas, USA / Austin (1970) | 2.58 birds ha^-1^ × 3418 g fw prey bird^-1^ breeding season^-1^ | 4.497 ^H^ | 0.337 | 4.834 |
| 90 | | Desert | Texas, USA / Austin (1970) | 1.5 birds ha^-1^ × 3418 g fw prey bird^-1^ breeding season^-1^ | 2.615 ^H^ | 0.196 | 2.811 |
| 91 | | Desert | Texas, USA / Austin (1970) | 2.03 birds ha^-1^ × 3418 g fw prey bird^-1^ breeding season^-1^ | 3.538 ^H^ | 0.265 | 3.803 |
| 92 | | Desert | California, USA / Austin (1970) | 4.72 birds ha^-1^ × 3418 g fw prey bird^-1^ breeding season^-1^ | 8.227 ^H^ | 0.617 | 8.844 |
| 93 | | Desert | California, USA / Austin (1970) | 6.35 birds ha^-1^ × 3418 g fw prey bird^-1^ breeding season^-1^ | 11.068 ^H^ | 0.830 | 11.898 |
| 94 | | Desert | Arizona, USA / Austin (1970) | 4.4 birds ha^-1^ × 3418 g fw prey bird^-1^ breeding season^-1^ | 7.669 ^H^ | 0.575 | 8.244 |
| 95 | | Desert | Arizona, USA / Austin (1970) | 5.4 birds ha^-1^ × 3418 g fw prey bird^-1^ breeding season^-1^ | 9.412 ^H^ | 0.706 | 10.118 |
| 96 | | Arctic tundra | Russia / Wielgolaski (1975) | 3 kg fw arthropods ha^-1^ year^-1^ | 3.00 |  | 3.00 |
| 97 | | Arctic tundra biome, wet areas | Russia / Wielgolaski (1975) | 9 kg fw arthropods ha^-1^ year^-1^ | 9.00 |  | 9.00 |
| 98 | | Arctic shrub tundra | Russia / Sokolov et al. (2012) | 0.90 birds ha^-1^ × 2093 g fw prey bird^-1^ breeding season^-1^ | 1.36 ^I^ | 0.10 | 1.46 |
| 99 | | Arctic shrub tundra | Russia / Sokolov et al. (2012) | 1.09 birds ha^-1^ × 2093 g fw prey bird^-1^ breeding season^-1^ | 1.64 ^I^ | 0.12 | 1.76 |
| 100 | | Arctic shrub tundra | Russia / Sokolov et al. (2012) | 2.45 birds ha^-1^ × 2093 g fw prey bird^-1^ breeding season^-1^ | 3.69 ^I^ | 0.28 | 3.97 |
| 101 | | Arctic tundra pond landscape | Canada / Montgomerie et al. (1983) | 0.70 birds ha^-1^ x 2093 g fw prey bird^-1^ breeding season^-1^ | 1.06 ^I^ | 0.08 | 1.14 |
| 102 | | Arctic tundra pond landscape | North America / James & Rathbun (1981) | 5.59 birds ha^-1^ x 2093 g fw prey bird^-1^ breeding season^-1^ | 8.42 ^I^ | 0.63 | 9.05 |
| 103 | | Arctic tundra | Canada / Watson (1963) | 1.25 birds ha^-1^ x 2093 g fw prey bird^-1^ breeding season^-1^ | 1.88 ^I^ | 0.14 | 2.02 |

^A^ Unless indicated otherwise, a nonbreeding season value is assumed to equal 7.5% of the corresponding breeding season value

^B^ 42% arthropods in diet

^C^ Energy utilized under field conditions equals 2.0 × EMR (existence energy) according to Väisänen & Järvinen (1977)

^D^ 59% arthropods in diet

^E^ 68.6% arthropods in diet

^F^ 16% arthropods in diet

^G^ During the breeding season, all bird species were insectivorous, with 95% arthropods in diet

^H^ Food consumption of a typical desert bird - cactus wren (weighing, on average, W = 38.9 g; Dunning 2007) – Standard Metabolism in kcal day^-1^ calculated with the equation SMR = 129 W^0.724^ of Lasiewski & Dawson (1967), whereby W is the average bird weight in kg; energy utilized under field conditions equals 2.5 × SMR (Holmes & Sturges 1975); “energy assimilated” converted to “prey consumption” taking into account a 75% assimilation efficiency (Wiens 1989). → 90-180 days (x̅ = 135 days) breeding season in deserts (Wiens 1991); bird densities taken from (Austin 1970). It is assumed that ≈60% of all desert bird individuals are insectivores and that arthropods make up ≈85% of the diet of the insectivores

^I^ Food consumption of a typical artic tundra bird – snow bunting (weighing, on average, W = 42.2 g; Dunning 2007) – Standard Metabolism in kcal day^-1^ calculated with the equation SMR = 129 W^0.724^ of Lasiewski and Dawson (1967), whereby W is the average bird weight in kg; energy utilized under field conditions equals 2.5 × SMR (Holmes and Sturges 1975); “energy assimilated” converted to “prey consumption” taking into account a 75% assimilation efficiency (Wiens 1989). → 100 days breeding season in tundra (Weiner & Głowaciński 1975); bird densities taken from (Watson 1963; James & Rathbun 1981; Montgomerie et al. 1983; Sokolov et al. 2012). It is assumed that arthropods make up ≈85% of the diet of the insectivores
